# Differential Expression of Immunogenic Proteins on Virulent *Mycobacterium tuberculosis* Clinical Isolates

**DOI:** 10.1155/2014/741309

**Published:** 2014-07-07

**Authors:** Pablo Schierloh, Laura Klepp, Camila Vazquez, Roxana Valeria Rocha, Federico Carlos Blanco, Luciana Balboa, Beatriz López, Viviana Ritacco, Fabiana Bigi, María del Carmen Sasiain

**Affiliations:** ^1^IMEX-CONICET, Academia Nacional de Medicina, Pacheco de Melo 3081, 1425 CABA, Argentina; ^2^Instituto de Biotecnología, CICVyA-INTA, N. Repetto and Los Reseros s/n, 1686 Hurlingham, Argentina; ^3^Servicio de Micobacterias, Instituto Nacional de Enfermedades Infecciosas ANLIS “Carlos G. Malbran”, Avenida Vélez Sarsfield 563, 1281 CABA, Argentina

## Abstract

Molecular epidemiology has revealed that *Mycobacterium tuberculosis* (*Mtb*), formerly regarded as highly conserved species, displays a considerable degree of genetic variability that can influence the outcome of the disease as well as the innate and adaptive immune response. Recent studies have demonstrated that *Mtb* families found worldwide today differ in pathology, transmissibility, virulence, and development of immune response. By proteomic approaches seven proteins that were differentially expressed between a local clinical isolate from Latin-American-Mediterranean (LAM) and from Haarlem (H) lineages were identified. In order to analyze the immunogenic ability, recombinant Rv2241, Rv0009, Rv0407, and Rv2624c proteins were produced for testing specific antibody responses. We found that these proteins induced humoral immune responses in patients with drug-sensitive and drug-resistant tuberculosis with substantial cross-reactivity among the four proteins. Moreover, such reactivity was also correlated with anti-*Mtb*-cell surface IgM, but not with anti-ManLAM, anti-PPD, or anti-*Mtb*-surface IgG antibodies. Therefore, the present results describe new *Mtb* antigens with potential application as biomarkers of TB.

## 1. Introduction

Increasing evidence suggests that genetic variation in* Mycobacterium tuberculosis* (*Mtb*) plays an important role in the outcome of the disease [1–3]. Owing to the lack of exchange of genetic material with a global microbial gene pool,* Mtb* had long been considered to have a clonal population structure. However, a significant genetic variation within* Mtb* has recently been unveiled by the identification of six phylogeographic lineages [2, 4, 5]. One of these lineages is the Euro-American clade, which includes all the spoligotype families predominating in the Western world, such as Haarlem, Latin-American-Mediterranean (LAM), and the not well-defined T group [6]. In particular, the Haarlem genotype is ubiquitous worldwide [7] and represents about 25% of the isolates in Europe, Central America, and the Caribbean [8]. In South America, the LAM family is the prevalent one, followed by the T and Haarlem families [9, 10]. Very recently, we had reported that in Buenos Aires area 37% of TB patients were infected with LAM, 34% with T and 17% with Haarlem lineages [11]. Recent epidemiological data suggest that differences in transmissibility and virulence among* Mtb* strains are related to the genetic background of the organism [12–14]. Particular outbreak strains were found to elicit distinct immune paths and mortality rates in the course of mice infection [15]. In this line, López et al. have demonstrated that mice infected with Beijing strains show accelerated bacterial multiplication, early and massive pneumonia, and death. Conversely, infection with Canetti strains induces a slow progressive disease characterized by delayed bacterial multiplication, limited pneumonia, steadily increasing granulomas, and virtually no mortality. In terms of virulence, strains belonging to two other conspicuous genotype families, the so-called Haarlem and Somali clades, displayed rather intermediate and less homogeneous results [14].

Antibody (Ab) responses strongly affect pathogenesis and outcome of disease caused by extracellular bacteria. However, Abs are also produced in infections caused by intracellular bacteria, where protective immunity is primarily elicited by the cell-mediated immune response [16]. In the case of tuberculosis (TB) the detection of circulating Abs in patients with active TB dates back to 1898 [17]. Even though an estimated 90% of TB patients produce Abs against* Mtb* [18], little is known about the correlation between their production, specificity, and disease process. The evaluation of Abs as biomarkers of active disease has been widely investigated [18–20]. However, due to the variation in Ab profiles among TB patients, their application as diagnostic tool has several limitations [18, 21, 22]. Moreover, host associated variables affecting Ab responses have been sporadically studied [23, 24]. Thus, despite much effort, we still do not know how much of the* Mtb* proteome is targeted by the human Ab response or how host characteristics and disease parameters affect target recognition.

As a classical model of intracellular pathogen, the immunity to* Mtb* is associated with a Th1-profile, in which the interleukin- (IL-) 12-interferon- (IFN-) *γ* axis together with tumor necrosis factor- (TNF-) -*α* is involved [25]. However, recent studies have revealed that* Mtb* developed a more complex host immune response [26]. It is well known that the cell envelope of* Mtb* is composed of proteins intermingled in a matrix of peptidoglycan, mycolic acids, lipids, and carbohydrates driving a complex and diverse structure which can influence the host immune response. We have previously demonstrated that inactivated clinical isolates from LAM and Haarlem families induced in vitro differential cytokine and reactive oxygen species (ROS) production by cells from innate and adaptive arms of the immune response in TB patients and healthy donors [27–29]. Moreover, the immune profiles seem to be associated with the families they belong to and not with a special characteristic of the isolate, suggesting that antigen composition might account for such differences [27–29].

The aim of the current study was to analyze whether the proteomic differences detected between the two clinical isolates could be reflected at the level of Abs response. We identified seven proteins that were differentially expressed between the local clinical LAM isolate LAM10406 and the Haarlem isolate H12425. Furthermore, Ab response against four of these proteins was detected in drug-sensitive and drug-resistant TB patients, showing cross-reactive humoral responses in most of the cases.

## 2. Materials and Methods

### 2.1. Patients

TB patients were recruited at the Servicio de Tisioneumonología, Hospital F. J. Muñiz, Buenos Aires, Argentina. Signed written informed consent was obtained according to Ethics Committee from Hospital F. J. Muñiz. Physical examination, serum extraction, complete blood cell count, electrolyte, sputum bacilloscopy, chest X-ray, and HIV test were carried out in the same institution. Exclusion criteria included positive HIV test, extrapulmonary dissemination of TB, and presence of concurrent infectious diseases. Inclusion criteria among TB patients were the presence of moderate to severe pulmonary involvement according to chest X-ray findings. Among enrolled TB patients (*n* = 37, average age = 32 yr, range = 21–55 yr), 18 had multi-drug-resistant (MDR) TB with severe pulmonary involvement, and among drug-sensitive TB, 11 had severe and 9 moderate pulmonary involvement. Bacteria from all MDR-TB patients were tested for drug-susceptibility and the genotype was determined by IS6110 DNA fingerprinting and spoligotyping according with standardized protocols [30, 31]. For drug-sensitive TB patients included herein, the infecting strain was not genotyped. Age matched No-TB patients were recruited at the Laboratorio Central-IHema, Academia Nacional de Medicina. Written informed consent was obtained according to Ethics Committee from the institutions. Exclusion criteria for No-TB patients included positive PPD-test, positive HIV-test, and familiar/occupational risk of contact with active TB patients. Inclusion criterion was serum immunoglobulin (Ig) G on normal reference range (700–1600 mg/dL). Among No-TB patients, 15 had idiopathic neutropenia (age range = 26–45 yr), 5 had thrombocytopenic disorders (age range = 36–51), 4 had blood malignant disorders (age range = 30–61 yr), 3 had fungal infection (age range = 35–43 yr), and 3 had Hepatitis C infection (age range = 25–41 yr).

### 2.2. Clinical* Mtb* Isolates

Clinical strains LAM10406 and H12425 are representative of prevalent LAM and Haarlem lineages and were obtained from sputum-culture-positive TB patients. Their genotype, drug-susceptibility, and the in vitro immune responses were previously described [27]. Cells were grown in Middlebrook 7H9 broth (Difco Laboratories, Detroit, MI, USA) at 37°C in 5% CO_2_ until exponential phase. For immunoassays, bacteria were killed by gamma irradiation at ≈1550 rads/minute during 27 hours (total dosage ≥ 2.5 Mrads) (CONEA, Argentina), suspended in pyrogen-free PBS at OD600 nm = 1 (~10^8^ bacteria/mL), and stored at −20°C until their use.

### 2.3. Analysis of the Proteomic Profiles of* Mtb* Strains

Pellets from 1 litre of 1-month-old cultures grown in Sauton medium of each strain were washed, resuspended in cold 1X PBS pH 7.4, 1 mM EDTA, 100X protease inhibitor mix (GE Healthcare), and disrupted using a Fast Prep FP120 bead-beater (40 s at 6 m s21, using Lysing Matrix B). The cell extract was clarified by centrifugation at 3000 ×g for 20 min and cell wall proteins were obtained by further centrifugation at 23000 ×g for 30 min. The supernatant containing the cell associated proteins was cleaned up with the 2D clean-up kit from GE Healthcare. Proteins from the cell wall pellet were obtained by the addition of 2 mL buffer (1X PBS pH 7.4, 1 mM EDTA, 2% SDS), followed by incubation for 2 h at 60°C and centrifugation for 15 min at 18000 ×g. Cell wall proteins present in the supernatant were then concentrated and desalted using again the 2D clean-up kit. The protein pellets were resuspended with rehydration buffer (8 M urea, 2% CHAPS, 0.5% IPG buffer pH 4–7, and 20 mM DTT), and 90 *μ*g protein in a final volume of 450 *μ*L was used to rehydrate a 24 cm immobilized pH 4–7 linear gradient strip (Immobiline DryStrips, GE Healthcare) for 16 h at room temperature, following the manufacturer's instructions. Isoelectric focusing was carried out with an Ettan IPGphor 3 system (GE Healthcare), using the following programme: 1, 0.5 kVh at 500 V; 2, 5.2 kVh (gradient) at 1000 V; 3, 13.5 kVh (gradient) at 8000 V; 4, 30 kVh at 8000 V. After focusing, the strips were equilibrated for 20 min in equilibrium buffer (2% SDS, 50 mM Tris/HCl, pH 8.8, 6 M urea, 30% glycerol, 0.002% bromophenol blue, and 0.5% DTT). The strips were then overlaid onto 12% SDS-polyacrylamide gels and, after electrophoresis, proteins were stained with the silver staining kit (GE Healthcare). Proteins from culture supernatants of* Mtb* strains were prepared as described previously [32] and resolved on 12% SDS-polyacrylamide gels, which were then stained with colloidal Coomassie (2 g Coomassie brilliant blue G250 l21; 0.5 g Coomassie brilliant blue R250 l21; 5% methanol, 42.5% ethanol, 10% acetic acid).

The differential bands or spots identified were cut from the gels, digested with modified porcine trypsin (Promega), and extracted as previously described [33]. Mass spectrometry (MS) analysis was carried out on a Bruker Ultraflex II mass spectrometer, with the matrix a-cyano-4-hydroxycinnamic acid. Data interpretation and protein identification were performed with the MS/MS spectra datasets using the MASCOT search algorithm (Version 1.6b9, Matrix Science, available at http://www.matrixscience.com/).

### 2.4. RT-qPCR

DNA-free RNA was extracted from 50 mL mid-exponential-phase cultures of strains grown in Middlebrook 7H9 medium supplemented with 0.05% Tween 80, as described by Santangelo et al. [34]. DNA-free RNA (1 *μ*g) was mixed with 50 ng of random primers (Invitrogen) in a final volume of 20 *μ*L and reverse-transcribed to total cDNA with SuperScript II reverse transcriptase (Invitrogen), following the manufacturer's instructions. Identical reactions lacking reverse transcriptase were also performed to confirm the absence of genomic DNA in all samples. RT-qPCR was performed in the* Applied Biosystems* 7000 DNA sequence detection system (Perkin-Elmer), with Master Mix QuantiTect SYBR Green (Qiagen), 1 *μ*L of template cDNA, and the pairs of primers listed in Table S1 in Supplementary Material available online at  http://dx.doi.org/10.1155/2014/741309. Each reaction was performed in duplicate. Results are presented as ratios calculated with the Relative Expression Software Tool (REST@) application described by Pfaffl et al. [35], based on four biological replicates for in vitro studies. Relative quantification of each target gene was performed by using* sigA* as a reference gene, with real-time PCR efficiencies of target and reference genes considered as 2. A subsequent test for significance of the results was performed by using the pair-wise fixed reallocation randomization test (http://rest.gene-quantification.info/).

### 2.5. Recombinant Proteins Expression and Purification

For immunogenic analyses, the differentially expressed proteins were cloned as glutathione-S-transferase (GST) fusions using the Gateway Cloning Technology (Life Technologies, USA). The genes were PCR amplified using chromosomal DNA from the strain in which they were identified, with the primers indicated in Supplementary Table S2 and cloned in the pENTR Directional TOPO (Invitrogen) to generate entry clones. LR-recombination reactions were carried out with LR Clonase II Plus enzyme mix (Invitrogen), following the manufacturer's instructions. Reactions were stopped by addition of Proteinase K and incubation at 37°C for 10 min. Destination expression vector pDEST15 was obtained from Invitrogen. The presence of the N-terminal GST tag in pDEST15 allows purification of recombinant fusion protein using glutathione agarose. The expression clones generated were used to transform BL21-AI (Invitrogen) competent cells. Recombinant* E. coli* were grown in 500 mL LB medium containing 125 *μ*g ampicillin mL^−1^ at 37**°C. When the OD_600_ reached 0.3–0.5, expression of the genes encoding the recombinant proteins was induced by addition of L-arabinose at final concentration of 0.2%. Cells were then harvested by centrifugation and resuspended in 100 mM Tris/HCl, pH**7.5, 1 M NaCl, 20% glycerol, and 1% NP-40. Soluble cell extracts from the cultures were prepared as described elsewhere [36] and recombinant proteins were purified from the supernatants by using Glutathione Sepharose (GE Healthcare), following the manufacturer's recommendation. Since we were not able to solubilize recombinant Rv2241 in the above conditions, its gene was transferred to the destination vector pDEST17 (Invitrogen) to be expressed as a His-fusion protein and purified under denaturing conditions using Urea 8 M. In this case the recombinant protein was purified from the supernatants by using Ni^+^ resin (Ni-NTA agarose, Qiagen), following the manufacturer's recommendation.

### 2.6. Indirect ELISA Assays

The following antigens (Ags) were employed: mannosylated lipoarabinomannan (ManLAM) from H37Rv strain (kindly provided by J. Belisle, Colorado State University, Fort Collins, CO, USA); protein purified derivative (PPD) (Statens Serum Institut, Copenhagen, Denmark). Homemade indirect enzyme-linked immunosorbent assay (ELISA) was used for detection of serum Abs employing goat anti-human polyvalent (anti-IgA/IgM/IgG) horseradish peroxidase- (HRP-) conjugated secondary Ab as detection reagent (Sigma-Aldrich Co. St. Louis, MO, USA). Briefly, 96-well high binding microplates (Corning, USA) were coated overnight (ON) at 4°C with 100 *μ*L of the following Ags: PPD (1 *μ*g/mL, in CO_3_ buffer pH = 9), ManLAM (0.5 *μ*g/mL, in PO_2_ buffer pH = 8), and recombinant proteins (1 *μ*g/mL, in PBS pH = 7.4). Optimal Ag concentration and coating buffer was experimentally determined (37, and data not shown). After properly washing with PBS 0.05% (v/v) tween-20 (Sigma) and blocking with PBS 2% (v/v) human serum albumin (HSA) (NatoCor, Córdoba, Argentina) plates were seeded and ON incubated at 4°C with serum dilutions: 1/1000 (PPD), 1/800 (ManLAM), and 1/500 (recombinant proteins). Serum dilutions were established in preliminary assays. Each sample was tested in duplicate. In assays with recombinant proteins (Figure 3), unspecific Abs were depleted from sera by adsorption on plates coated with glutathione-S-transferase (GST) (1 *μ*g/mL, in PBS pH = 7.4) for 1 h at 37°C before serum-Ag incubation step. To deplete Abs directed to surface exposed* Mtb-*Ags from sera samples (Figure 5(b)), 150 *μ*L of 1/500 diluted sera was also preadsorbed with 10^6^ *γ*-irradiated H12435 or LAM10406 clinical isolates for 1 h at 37°C before serum-Ag incubation step. Finally, secondary reagent (100 *μ*L, 1/3000 dilution) was added for 30 min and, after extensive washing, HRP activity was measured using tetramethylbenzidine (TMB) substrate (Sigma). Absorbance was measured at 450 nm (subtracting 570 nm background signal) using an UVM340 plate reader with DigiRead software (Asys Hitech, UK).

### 2.7. Flow Cytometry Assays

Serum Abs directed to surface exposed* Mtb-*Ags were determined by flow cytometry (FACS) analysis. Briefly, 10^6^ *γ*-irradiated LAM10406 were incubated for 30 min at room temperature (RT°) with 1/500 serum dilution, washed 2 times with PBS-HSA 2% by centrifugation at 800 ×g for 5 min, and stained with anti-human(h)IgM-FITC (polyclonal goat *μ* chain-specific, 1/1000 dilution, Cappel) or anti-hIgG-PE (mouse monoclonal anti-human Fc-*γ*, 1/600 dilution, Becton Dickinson). Data acquisition of stained bacteria was performed immediately using log-FSC/log SSC amplification mode in a FACScan cytometer and CellQuest software (BD). In order to estimate the isotype of Abs, a latex bead based FACS assay was employed. Briefly, each recombinant protein (~200 *μ*g/mL) was passively adsorbed to latex beads (*Ø* = 3 *μ*m, 1% v/v, Sigma) by incubating in 3-(N-morpholino) propanesulfonic acid (MOPS) buffer (40 mM, pH = 6.5) for 3 h at RT° with gentle agitation (100 rpm). Then, protein-coated beads were extensively washed with 2% PBS-HSA and incubated with 1/400 diluted GST-preadsorbed serum for 30 min at RT°. Finally, after 2 washings, beads were stained with anti-hIgM-FITC, anti-hIgG-PE, and anti-hIgA-FITC (polyclonal goat *α* chain-specific, 1/1000 dilution, Cappel).

### 2.8. Data Management, Statistics, and Immunoinformatics

All figures were edited with Photoshop (Adobe, USA). Excel (Microsoft, USA) was employed for storage, transformation, compilation, and edition of data sets and for depicting the figures' heat-maps and tables. FACS data analysis was performed with FCS express 4 (DeNovo Software, CA, USA). Figures' graphs and uni/bivariate statistical analyses were performed using Prism5 (GraphPad, USA) assuming a *P* < 0.05 as significant. SPSS 15.0 (IBM, USA) was employed for multivariate statistical analysis. Protein structure homology models were computed by SWISS-MODEL web server (SIB, Switzerland) [37]. Best rated protein 3D structure models were energy relaxed by mean of normal mode-based molecular dynamics simulations using the NMSim web server (CPC, Germany) [38]. B-cell epitope predictions based on crystallographic (Rv0009, Rv0407) or modeled 3D protein structures (Rv2241, Rv2624c) were accessed with DiscoTope 2.0 server (CBS, Denmark) [39]. PyMOL (Delano Scientific LLC, USA) was employed to depict predicted epitopes on each molecule (see Supplementary Table S3).

## 3. Results and Discussion

### 3.1. Identification of Expressed Proteins by LAM10406 and Haarlem (H) 12425 Clinical Isolates

**Figure 1:**
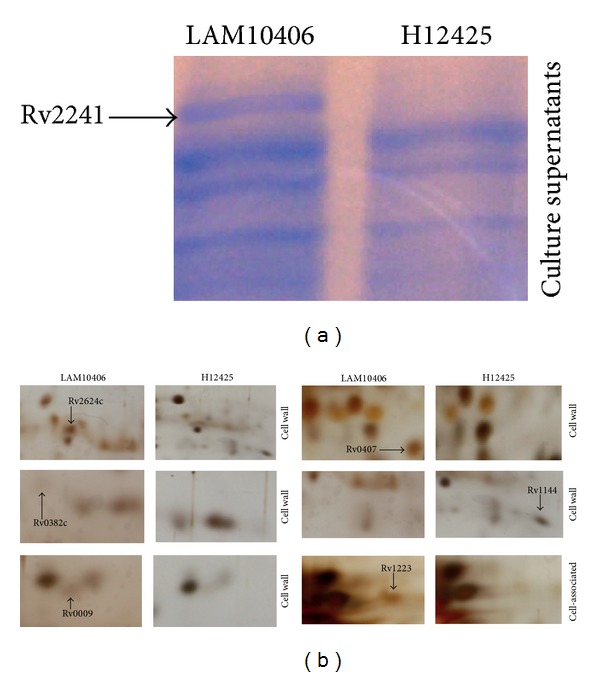
Proteomics to identify differential proteins between LAM10406 and H12425* M*.* tuberculosis* strains. (a) Equal amounts of proteins from culture supernatants of* Mtb* LAM10406 and H12425 were resolved on 1D 12% polyacrylamide gels, which were stained with colloidal Coomassie. The identified differential protein is indicated with the arrow. (b) Differential proteins identified by 2D gel electrophoresis. The first-dimension isoelectric focusing used a pH range of 4–7. The second-dimension SDS-PAGE was on 12% polyacrylamide gels, which were silver stained. Differential proteins present in the cell wall and cell-associated fractions of the* Mtb* LAM10406 and H12425 strains are indicated.

The rationale behind searching for differentially expressed proteins between a strain belonging to LAM and a strain to Haarlem families was to find proteins that could explain the differential innate/adaptive immune responses developed by these two families [26–28]. To identify these proteins we compared the protein expression patterns of the H12425 and LAM10406 strains by 1D- and 2D-PAGE. In a first approach we prepared proteins from culture filtrates of both strains and resolved them by 1D electrophoresis gels, with subsequent colloidal Coomassie staining. In this case, we identified one differential band (Figure 1(a)) that was overexpressed in LAM10406. This band was cut from the gel and trypsin-digested to be identified by mass spectrometry (MS). Then, we analyzed the cell wall and cell associated protein fractions by silver-stained 2D electrophoresis gels. The comparison of two sets of 2D gels from each strain revealed four proteins present in the cell wall fraction of LAM10406 and absent in H12425, one protein from the same fraction present in H12425 and absent in LAM10406 and one protein only present in the cell associated fraction of LAM10406 (Figure 1(b)). All these proteins were identified by mass spectrometry and the results are summarized in Table 1. Although the functions of the proteins between both strains are diverse, most of them localize in the cell wall, where they may trigger a differential host immune response, which could impact the outcome/progression of the disease.

**Figure 2:**
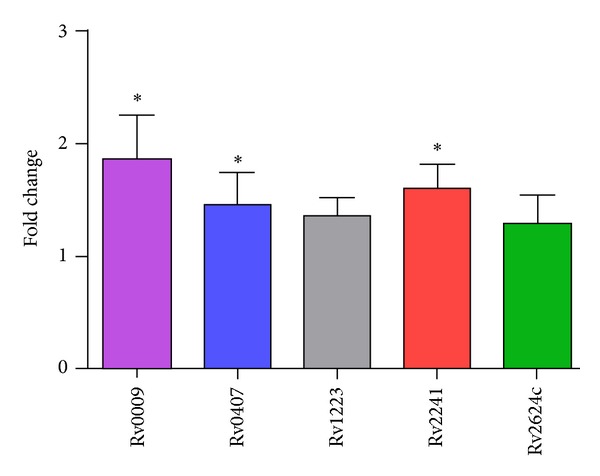
Gene expression fold-change differences between LAM10406 and H12425 using RT-qPCR. Relative gene expression was calculated using the 2-ΔΔCt method with E correction, using sigA mRNA expression as reference genes and H12425 as the calibrator. Data were analyzed using a random permutation test (*P < 0.05). The bars indicate the average ratios of LAM10406/H12425 ± SD.

The differential expression of the genes encoding the identified proteins was then analyzed by RT-qPCR. Of the seven proteins, the overexpression of five of them in LAM10406 could be validated at the transcriptional level (Figure 2). The regulation in gene expression in the nonvalidated cases could occur at a posttranscriptional level.

Then, we analyzed whether the proteins were involved in the immune response triggered by the two* Mtb* families. To this end, we cloned and expressed the RT-qPCR validated genes in* Escherichia coli*, by using the Gateway technology. Since one of the validated genes failed our attempt at PCR amplification (Rv1223), we continued working only with the remaining four proteins (Rv2624c, Rv0009, Rv0407, and Rv2241). All the proteins were cloned as GST fusions with the exception of Rv2241, which was expressed as a His-fusion protein and purified under denaturing conditions because of its insolubility under other conditions.

### 3.2. Rv2241, Rv0407, Rv2624c, and Rv0009 Induce Antibody Responses on Subsets of Drug-Sensitive and MDR-TB Patients

**Figure 3:**
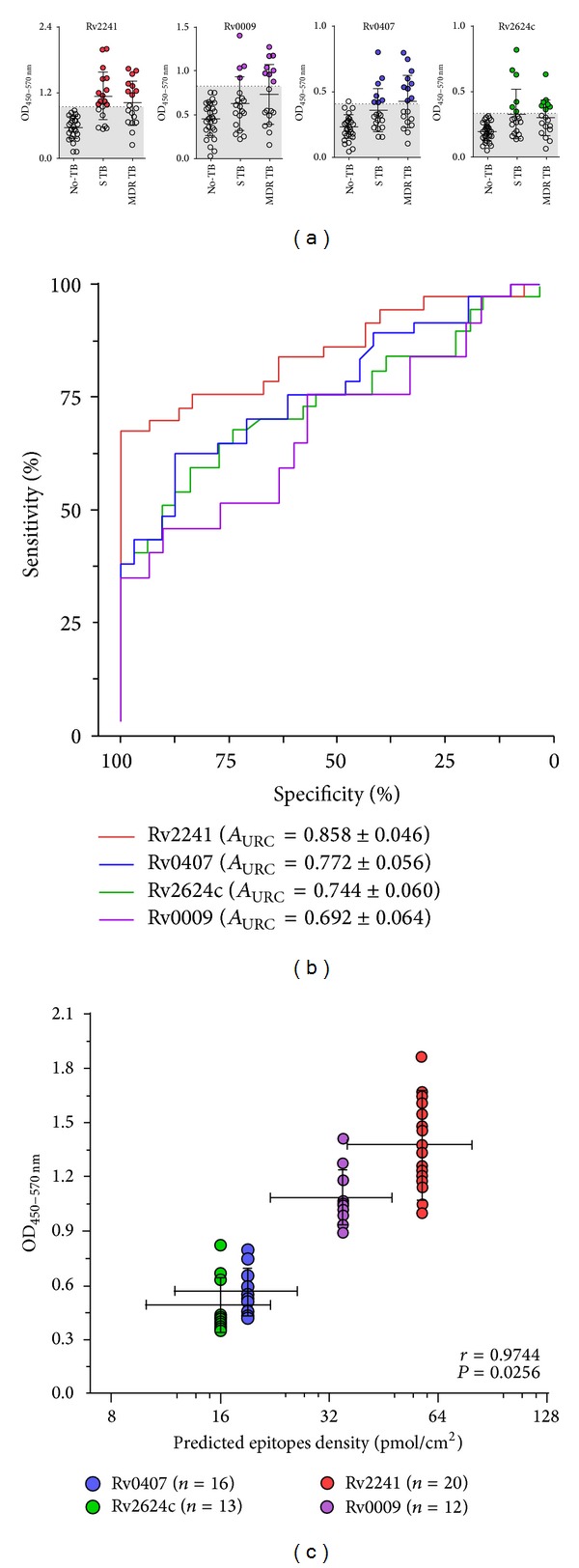
Antibodies against Rv2241, Rv0009, Rv0407, and Rv2624c recombinant proteins in drug-sensitive and MDR-TB patients' sera. ELISA plates were coated with recombinant proteins, loaded with 1/500 serum dilutions, and reveled with HRP conjugated anti-human polyvalent IgM/A/G. (a) 1D scatter plots indicating serological results of No-TB (No TB, *n* = 31), drug-sensitive TB (S TB, *n* = 19), and multi-drug-resistant TB (MDR TB, *n* = 18) patients' sera. Threshold, defined by maximum likelihood ratio (ROC analysis), was shaded in gray. Seropositive samples for each protein were color highlighted (Rv2241 = red, Rv0009 = purple, Rv0407 = blue, and Rv2624c = green). (b) ROC analysis indicating area under curve ± 95% CI. (c) Correlation analysis between signals (mean ± SEM) of positive samples versus immune-informatics predicted epitope density (median ± range) for each recombinant protein.

Purified recombinant proteins were used as coating antigens for serum ELISA assays. As shown in Figure 3(a), circulating Abs against all these proteins were detected in subsets of TB patients. Anti-Rv2241 Abs were the more frequently found (~54%), followed by anti-Rv0407 (~43%), anti-Rv2624c (~35%), and anti-Rv0009 (~32%). Receiver operating characteristic (ROC) analysis demonstrated that immune reactivity against all of these proteins can statistically distinguish the TB patient population from the No-TB population (Rv2241: *P* < 0.0001; Rv0407: *P* < 0.0002; Rv2624c: *P* < 0.0006; Rv0009: *P* < 0.0073). However, regarding clinical value of these Ags in serological diagnosis of pulmonary TB, only Rv2241 exhibited a moderate performance (% area under ROC curve ≥ 85%, Figure 3(b)). Besides, we did not find significant association between drug-susceptibility status (drug-sensitive TB versus MDR-TB), Mantoux test (PPD^+++^ versus PPD^+^ versus PPD^neg^), and the pulmonary involvement (Moderate versus Severe) with seroreactivity for any of the selected proteins (*P* > 0.05; *χ* ^2^-tests with Yates' correction). Interestingly, according to the interrogation of a database that contains published epitopes (http://www.iedb.org/; our last query = 5/23/14), none of the above indicated proteins have been previously reported as immunogenic/antigenic with the exception of Rv2241. Particularly, two 9-mer peptides present in Rv2241 sequence bind with high affinity to human HLA-I molecules [40], which potentially can induce T-cell dependent immune responses.

In terms of signal-to-noise ratio of ELISA assays, strong differences among each recombinant protein were detected. Given that this may affect accuracy and sensitivity of the test, we evaluated if protein specific characteristics might account for such differences. The theoretical range of Ab binding sites per well was estimated (see Supplementary Table S3). For this purpose several factors were considered: protein structure-based immunoinformatic predictions, the saturating protein-binding capacity of microplates, and the molecular weight (Mw) of the recombinant fusion proteins. As it is shown in Figure 3(c), a positive correlation between predicted epitope density for a given recombinant protein and its corresponding positive ELISA signal was verified (OD_450 nm–570 nm_ values from seropositive samples). This result suggests that structural characteristics of the proteins may explain variations in the signal/noise ratio output. Furthermore, this result may be useful for introducing technical improvements to the assay.

Having observed that Rv2241, Rv0407, Rv2624c, and Rv0009 are augmented at mRNA and protein levels in axenic cultures of LAM10406 strain (Table 1), we wondered whether an association between Abs against these proteins in sera from TB patients and the infecting strain could be detected. To assess this we must restrict our analysis to MDR TB patients given that genotyping data of infecting strains for drug-sensitive TB patients were not available. Interestingly, all patients infected with non-LAM strains (Haarlem *n* = 7; Tuscani *n* = 1) were seroreactive against at least one recombinant protein. On the other hand, only two samples among LAM-infected patients (*n* = 10) were seropositive (Table 2). These results showed a statistical association between non-LAM infections and immunoreactivity (*P* = 0.0035; *χ* ^2^-tests with Yates' correction), which is in contrast to the presumption expected. Any possible explanation behind these apparently conflicting data could only be achieved after analyzing a greater number of locally circulating* Mtb* strains at proteomic level and a larger serological survey that should include not only MDR-TB patients but also drug-susceptible TB patient infected with known lineage strains.

### 3.3. Antibody Responses against Rv2241, Rv0009, Rv0407, and Rv2624c Are Mutually Correlated but Independent of the Presence of Anti-PPD or Anti-ManLAM Antibodies

**Figure 4:**
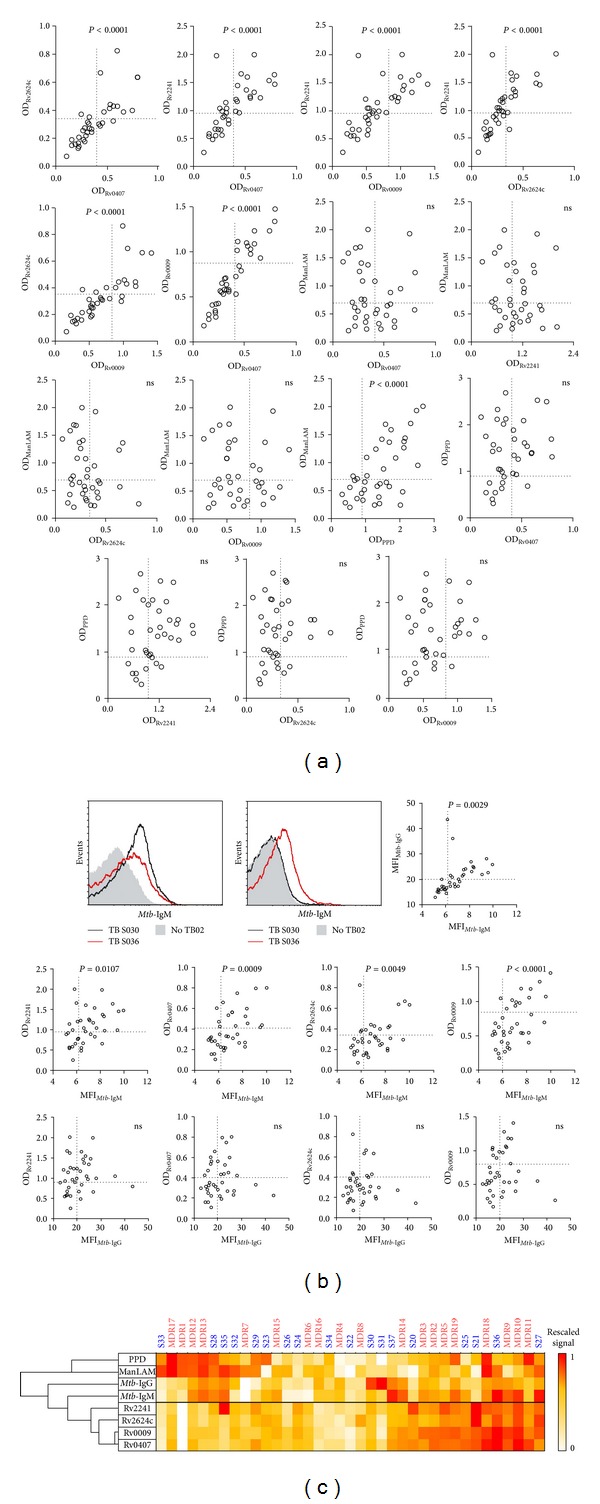
Cross-reactivity analysis of humoral immune responses in drug-sensitive and MDR-TB patients' sera. (a) 2D scatter plots of combinatorial correlations among results against protein-specific and complex Ags in TB patients' serum samples (*n* = 37). Pearson correlation test was applied after verification of normal distribution of all data sets. “ns” indicates *P* > 0.05. Thresholds are delimited by dotted lines. (b) FACS analysis of cell surface binding-IgM and -IgG to LAM10406 in sera from TB patients (*n* = 37). Histogram plots of two TB and one No-TB patients are showed for comparison. Correlation between median fluoresce intensity (MFI) and protein-specific Abs (Pearson correlation test). “ns” indicates *P* > 0.05. Thresholds are delimited by dotted lines. (c) Antigens were classified as function of their pattern of seroreactivity in the 37 TB patients (hierarchical clustering based on the correlation). Matrix of 0 to 1 rescaled values is represented as heat-map.

One interesting observation from Table 2 is the abundance of MDR-TB patients' sera that react with more than one recombinant protein. In order to test if cross-reactivity is confined to recombinant proteins and if it is a specific feature of MDR-TB patients, we extended our analysis to other* Mtb*-Ags and to drug-sensitive TB patients. As can be observed in (Figure 4(a)), a strong correlation in Ab titers among each of the recombinant proteins was observed, but not with PPD or ManLAM Ags. Next, we tested if IgM and IgG Abs that bind* Mtb*-cell surface exposed structures were correlated with those directed to recombinant proteins. As it is shown in Figure 4(b), Ab titers against proteins correlated with anti-*Mtb* IgM but not with anti-*Mtb*-IgG. By multivariate based hierarchical classification, we concluded that Rv0407, Rv0009, Rv2624c, Rv2241, and* Mtb*-IgM Ags may be clustered together as function of their seroreactivity (Figure 4(c)). Analyses of antigen recognition by individual sera revealed that the pattern of antigens reacting with serum Abs varied greatly from patient to patient; thereby, no clear division among patients with MDR versus drug-sensitive as well as with bacillary loads was observed (Table 2).

### 3.4. Isotype Analysis of Anti-Rv2241, Rv0009, Rv0407, and Rv2624c Antibodies

**Figure 5:**
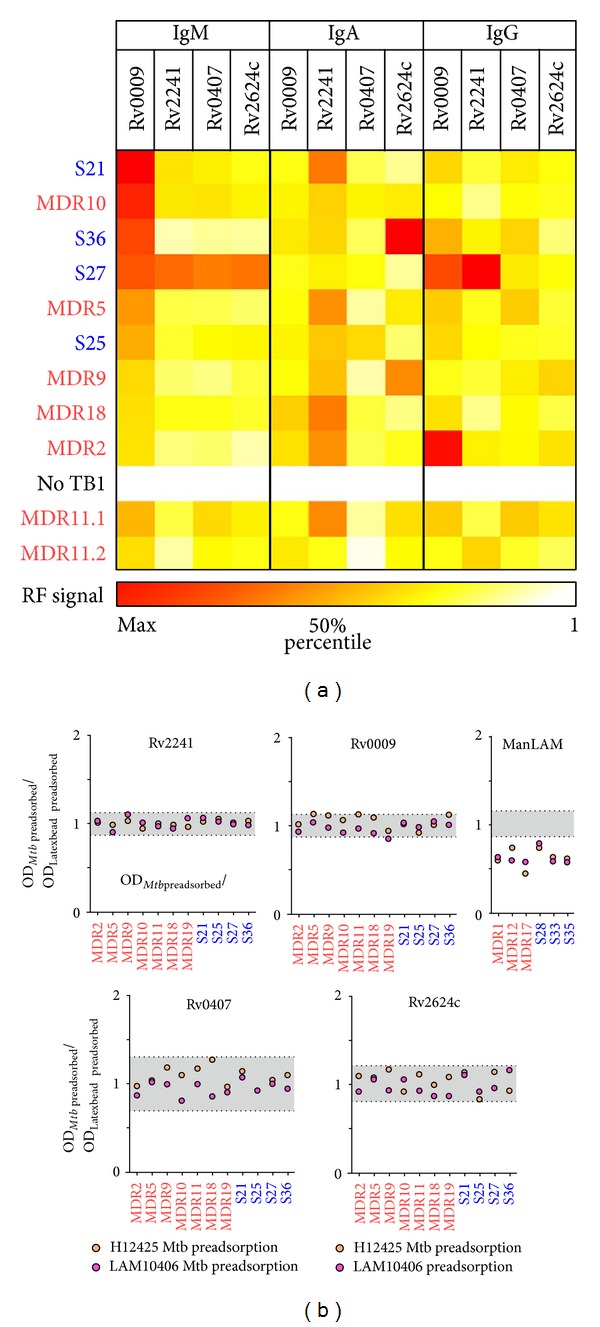
Characterization of anti-Rv2241, Rv0009, Rv0407, and Rv2624c antibodies from seropositive patients. (a) Isotype characterization of anti-Rv2241, Rv0009, Rv0407, and Rv2624c binding Abs was performed by FACS analysis using latex bead as Ag adsorbing solid support. For data normalization, MFI values of seropositive TB patients (*n* = 10 + 1 repeated sample/MDR11.2) were divided by MFI values of a No-TB patient (No TB1). Matrix of values was represented as heat-map. (b)* Mtb*-cell surface recognition by anti-Rv2241, Rv0009, Rv0407, and Rv2624c Abs was evaluated by preadsorbing seropositive (*n* = 11) samples with *γ*-irradiated whole LAM10406 and H12425* Mtb* cells or with HSA-Latex Beads (negative binding control) before incubation step of ELISA assay. Results were presented as ratio between* Mtb* preadsorbed over mock preadsorbed OD data. Range of unspecific/experimental variation (gray shaded areas) was estimated by ratio between replicates. For comparison, binding inhibition to ManLAM was performed in parallel using selected ManLAM-seropositive samples (*n* = 5).

The functionality of Abs is largely determined by the specific interaction of their constant fragment (Fc) domain with host cell Ig receptors. To better describe the circulating populations of anti-Rv2241, Rv0009, Rv0407, and Rv2624c Abs, we performed an immunoisotype characterization based on an Ag-specific FACS analysis [41]. To do this, sera reactive against all the recombinant proteins (*n* = 10) were incubated with recombinant protein-coated latex beads (Figure 5(a)). Because different structural features of proteins may affect latex bead coating efficiency as well as the employment of different fluorescent-labeled secondary reagents, this assay only allows vertical comparisons (between patients). However, within a given isotype group, Rv0009-specific IgM and Rv2241-specific IgA tended to be more abundant than the others. Besides, we were able to compare two samples from the same TB patient, one taken early when he was impatient (MDR11.1) and the other taken later on when the clinical symptoms declined at the moment of hospital discharge (MDR11.2). We observed that the diminution in Rv2241-specific IgM and IgA was accompanied by an increase in Rv2241-specific IgG, which is consistent with a class-switching event. However, these observations will need further confirmation in more extended studies.

### 3.5. *Mtb*-Surface Accessibility of Rv2241, Rv0009, Rv0407, and Rv2624c Antigenic Determinants

The outcome of mycobacterial infections is thought to be critically dependent on the nonopsonic invasion and colonization of macrophages during primary infection of inhaled bacteria. Indeed, if* Mtb* is opsonized by cell wall specific Abs, it becomes more susceptible to microbicidal action of phagocytes and complement system [42]. Herein, we observed that* Mtb*-surface binding IgM was correlated with Abs against Rv0009, Rv0407, Rv2624c, and Rv2241 (Figure 4(b)) and that, excluding the later, the three are* Mtb* cell wall associated proteins (Table 1). Given these results, we assessed if the Abs that recognized those proteins were also able to bind surface-exposed structures on whole bacilli. For this purpose, selected seropositive samples were preadsorbed with clinical isolates (LAM10406 and H12425) or mock preadsorbed (HSA-coated Latex Beads) before ELISA assay. In order to show that this indirect assay is sufficiently sensitive to reveal the presence of anti-surface Abs that also bind to a given Ag, we employed ManLAM, a well characterized* Mtb*-cell surface, that exposed Ag as positive control. We observe that preadsorption of anti-ManLAM-positive sera (*n* = 6) with both clinical* Mtb* isolates reduced the specific binding to ManLAM when compared with mock preadsorbed samples (Figure 5(b)). By contrast, Ab depletion by preadsorption of 11 antiprotein positive sera with both* Mtb* strains was not able to reduce the immunoreactivity against nonrecombinant protein (Figure 5(b)). Altogether, the above results indicate that surface-exposed epitopes on LAM10406 and H12425 cells were not efficiently/sufficiently recognized by anti-Rv0009, Rv0407, and Rv2624c Abs, suggesting that these Igs have limited or null* Mtb*-opsonic capacity. Moreover, we observed the same results when we tested anti-Rv2241 Abs, which are expected to bind secreted epitopes (Table 1).

## 4. Conclusions

Based on the comparison of two locally circulating* Mtb* clinical isolates that exhibited divergent stimulatory properties on human leukocytes [27–29], herein we identify seven differentially expressed proteins. Four of these proteins were overexpressed in cultures of LAM10406 strain and they are able to induce humoral immune response in the human host during natural infection. Unexpectedly, when the infecting genotype was correlated with serology, a higher proportion of seropositive cases among patients infected with no LAM-strains were observed. Interestingly, those TB patients that exhibit seroreactivity against one protein also show substantial cross-reactivity against the others, suggesting that these proteins are coexpressed during infection. However, in order to be confirmed, extended, and/or explained, all these interesting observations will require further studies involving a greater number of clinical isolates from different linages and a more representative cohort of TB patients.

Rv2241 was the most immunogenic protein studied, with some potential in terms of practical application as biomarker. Interestingly, Rv2241 is a secreted protein, a feature that has been previously highlighted regarding humoral immune responses in human TB [21–24]. On the other hand, the specific Abs against the cell-wall associated proteins Rv0407, Rv2624c, and Rv0009 seem to have low opsonic capacity, which might be ascribed to the complex structure and chemical composition of the mycobacterial cell wall.

Additionally, given that in serological studies the bioinformatics approaches are only marginally used, we consider that the successful application of a B-cell epitope prediction tool to explain our experimental outputs is* per se* an interesting finding. For example, similar strategies may be followed during rational design of more sensitive Ag-immobilized based immunoassays.

The humoral immune response in human TB has been shown to be highly variable and strongly influenced by multiple factors. This reinforces the idea that combination of classical immunochemical assays with modern approaches can give a better understanding of such complexity. In this line, the present study makes use of the information provided by genetic, proteomic, and immunoinformatic outputs in order to contextualize the noisy data that come from serological analysis of TB patients.

## Supplementary Material

Supplementary Material is constituted by three tables. Supplementary tables S1 and S2 provide nucleotide sequences of primers used along the present work. Table S3 provide information regarding bioinformatics-predicted B-cell epitopes of recombinant proteins used in serological studies.

## Figures and Tables

**Table 1:** Differentially expressed proteins between *M. tuberculosis* LAM10406 and *M. tuberculosis* H12425 identified by 1D and 2D gels.

Rv number	Gene symbol	Description∗	Cellular localization	Strain in which it is overexpressed
Rv2624c	None	Conserved hypothetical protein	Cell wall	LAM10406

Rv0382c	pyrE	Probable orotate phosphoribosyltransferase PyrE	Cell wall	LAM10406

Rv0009	ppiA	Probable iron-regulated peptidyl-prolyl cis-trans- isomerase A PpiA	Cell wall	LAM10406

Rv0407	fgd1	Probable F420-dependent glucose-6-phosphate dehydrogenase Fgd1	Cell wall	LAM10406

Rv1144	None	Probable short-chain type dehydrogenase/reductase	Cell wall	H12425

Rv1223	htrA	Probable serine protease HtrA	Cell associated	LAM10406

Rv2241	aceE	Probable pyruvate dehydrogenase E1 component AceE	Culture supernatant	LAM10406

*According to TubercuList (http://genolist.pasteur.fr/TubercuList/).

**Table 2:** Immunoreactivity of TB MDR patients infected with Haarlem, LAM, and Tuscani *Mtb* lineages.

Patient data					Serology^2^			
Code	Sex	Age	SPT family^1^	BAAR	Rv2241	Rv0407	Rv0009	Rv2624c
MDR18	F	24	Haarlem	+	+++	+++	+++	+
MDR5	F	35	Haarlem	−	+++	++	+	++
MDR19	M	28	Haarlem	+++	++	++	++	+
MDR11	M	28	Haarlem	+	+	+++	+	+
MDR2	F	44	Haarlem	+	+	+	++	+
MDR3	F	47	Haarlem	+	+	+	++	−
MDR14	F	35	Haarlem	−	−	+	−	−

MDR8	F	27	Tuscani	−	−	−	−	+

MDR10	F	23	LAM	+	+++	+++	+++	+++
MDR9	F	20	LAM	−	++	++	+++	+
MDR6	M	49	LAM	−	−	−	−	−
MDR12	M	28	LAM	−	−	−	−	−
MDR17	F	46	LAM	+++	−	−	−	−
MDR7	F	36	LAM	+	−	−	−	−
MDR13	M	24	LAM	+++	−	−	−	−
MDR4	F	40	LAM	+	−	−	−	−
MDR16	F	48	LAM	+++	−	−	−	−
MDR1	F	42	LAM	+	−	−	−	−

^1^For each MDR TB patient, genotype of infecting strain was assessed by standard spoligotyping methods [30, 31]. ^2^The relative OD values among seropositive samples are represented by “+++” (high OD values), “++” (mid OD values) and “+” (low OD values). Seronegative samples are indicated by “−” (OD values below cutoff measure).
