# Supplementary material for: Differential Expression of Immunogenic Proteins on Virulent Mycobacterium tuberculosis Clinical Isolates
Source: Biomed Res Int. 2014 Jul 7;2014:741309. doi: 10.1155/2014/741309 (PMC4109345; doi:10.1155/2014/741309)
Supplement: Supplementary file 1 — Supplementary Material is constituted by three tables. Supplementary tables S1 and S2 provide nucleotide sequences of primers used along the present work. Table S3 provide information regarding bioinformatics-predicted B-cell epitopes of recombinant proteins used in serological studies. [file 741309.f1.pdf]

Supplementary table S1: Primers used in RT-qPCR studies.

Gene	Forward primer (5´–3´)	Nt position[1]	Reverse primer (5´–3´)	Nt position[2]
Rv2624	agccgacgatgaaaacaatc	16	atgtgtcggcttgatcactg	150
Rv0382c	cgactgggactattcggttg	176	ccttcgataagtcgctgcat	323
Rv0009	gacatcaagatcgccctgtt	72	atgcgttttgggtcgaatag	172
Rv1144	catcaacctagtcggcacct	323	aggcggtgttaatgatgacg	427
Rv0407	ccgtcgaaaaggtaggtcaa	877	cgactggaagagctccagaa	978
Rv1223	cgatcaaccacggtaactcc	1141	gccaccaatttcatctcgtt	1283
Rv2241	gagcgtggatcagatggaat	241	gtggttgaaaccgacctcat	366

[1]refers to the position in the gene of the 5´ base of the forward primer

[2] refers to the position in the gene of the 5´ base of the reverse primer

Supplementary table S2: Primers used to amplify the validated genes.

Gene	Forward primer (5´–3´)	Reverse primer (5´–3´)
Rv2624c	caccatgtctgggagaggagagcc	tcagcggcgaacgacaagca
Rv0009	caccatggcagactgtgattccgt	tcaggagatggtgatcgact
Rv0407	caccgtggctgaactgaagctagg	tcagccaagtcgccgcaacc
Rv2241	ttaggccccgggaccgggatccgtg	caccgtggcgtcgtatttgcccgacattg
Rv1223	caccgtggatactagggtggacac	ctaggtgctatcggggtccg

Supplementary table S3: B-cell epitope prediction

Protein	Crystal Structure	3D Model Template[1]	DP[2]	Figure[3]	Predicted Epitope density[4] (pmol/cm^2)
**Rv0009**<br>Iron-regulated peptidyl-prolyl cis-trans isomerase A **PpiA**	PDB id: 1W74<br>Chain: A<br>BU: Monomer		**38**<br>(171)	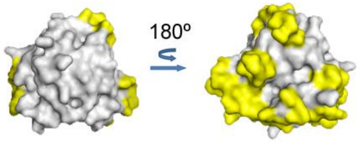	**35±13**
**Rv0407**<br>f420-dependent glucose-6-phosphate dehydrogenase	PDB id: 3B4Y<br>Chains: A-B<br>BU: Homodimer		**28**<br>(333)	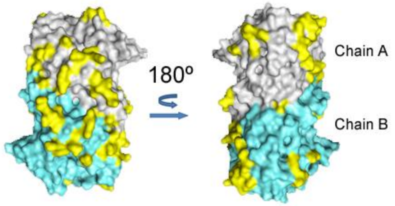	**19±7**
**Rv2241**<br>Pyruvate dehydrogenase E1 component **AceE**	NA	PDB id: 1L8A<br>Chain: A<br>BU: Homodimer	**173**<br>(895)	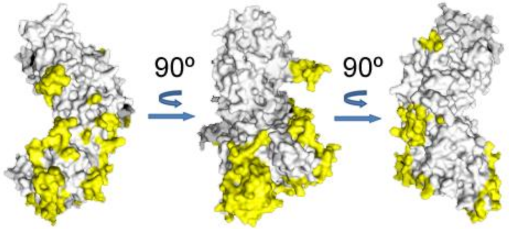	**58±22**
**Rv2624c**<br>Hypothetical stress protein	NA	PDB id: 1MJH<br>Chain: A<br>BU: Homodimer	**21**<br>(279)	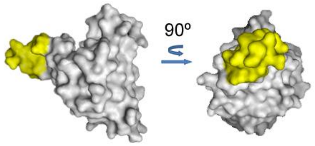	**16±6**

[1]For those proteins with no published 3D structures (NA), we performed structure homology modeling using the SWISS-MODEL server, an on-line free-access tool (http://swissmodel.expasy.org/workspace). The Protein Data Bank identification (PDB id) of template structure used is indicated.
[2]Discotope prediction (DP) were residues with scores >-3,7 determined by DiscoTope 2.0 algorithm. Total # residues on protein 3D structure were indicated between brackets. DiscoTope 2.0 server is a free access prediction tool (http://www.cbs.dtu.dk/services/DiscoTope-2.0/).
[3]Predicted discontinuous B cell epitopes were highlighted in yellow on the published or modeled 3D structures. Molecules were depicted using the PyMOL software.
[4]To estimate the predicted surface density of binding sites per ELISA microplate wells, we considered the following parameters: (1) Number of Discotope predicted aminoacidic residues per molecule; (2) Mw of Rv protein+ Mw of GST/HIS-tag; (3) Maximal protein binding capacity of Microplate (Corning High Binding Polystyrene)=500 ng/cm^2 ; (4) Based on published data, one conformational epitope involve between 8-22 aminoacids (95% CI= 8.6-19). The table informed value is Median±Range. Min and Max range values were calculated by considering 19 and 8.6 aminoacids /epitope, respectively.
